# Association between commercial funding of Canadian patient groups and their views about funding of medicines: An observational study

**DOI:** 10.1371/journal.pone.0212399

**Published:** 2019-02-15

**Authors:** Joel Lexchin

**Affiliations:** 1 School of Health Policy and Management, York University, Toronto, ON, Canada; 2 Emergency Department, University Health Network, Toronto, ON, Canada; 3 Department of Family and Community Medicine, University of Toronto, Toronto, ON, Canada; Universita degli Studi di Padova, ITALY

## Abstract

**Background:**

Patient groups represent the interest of their members when it comes to drug funding. Many patient groups receive grants from pharmaceutical companies that make products being considered for funding. This research examines whether there is an association between the positions that Canadian groups take about the products and conflicts of interest with the companies.

**Methods:**

The Common Drug Review (CDR) and panCanadian Oncology Drug Review (pCODR) make recommendations to Canadian provincial and federal drug plans about funding particular drug-indications. Both utilize input from patient groups in making their recommendations. Patient group submissions are available from both organizations and these submissions contain statements about conflicts of interest. Views of the patient groups, with and without a conflict with the company making the drug under consideration and without any conflicts at all, were assessed and then compared with the recommendations from CDR and pCODR.

**Results:**

There was a total of 222 reports for drug-indications. There were 372 submissions from 93 different patient groups. Groups declared a total of 1896 conflicts with drug companies in 324 (87.1%) individual submissions. There were 268 submissions where groups declared a conflict with the company making the product or said they had no conflict. Irrespective of whether there was a conflict, the views of patient groups about the drug-indications under consideration were the same. There was no statistically significant difference between views of patient groups and the recommendations from CDR and/or pCODR.

**Conclusions:**

The large majority of patient groups making submissions about funding of particular drug-indications had conflicts with the companies making the products and their views about the products were almost always positive. This association between funding and views needs to be further investigated to determine if a true cause and effect exists.

## Introduction

Patient groups have been set up in Canada to represent patients at multiple levels in the healthcare system and more widely. Typically, they are concerned with people suffering from a single condition, but they may also represent people with multiple related conditions, e.g., various forms of arthritis. They may lobby for Health Canada to approve new drugs and for particular products to be provided for their members. They also speak for patients with healthcare professionals and healthcare institutions such as hospitals and finally they are often the voice of patients in the media.

Since the Canadian federal government rolled back funding of patient groups in the mid 1990s [1] groups have had to seek new sources of revenue and many of them receive money from pharmaceutical companies. Some groups have lobbied provincial governments to include drugs on formularies made by companies from which they receive grants [2, 3].

There has not been any systematic analysis of patient group funding by pharmaceutical companies in Canada and whether there is an association between funding and the activities of groups. This study looks at this issue by examining the submissions that patient groups make to the Common Drug Review (CDR) and the panCanadian Oncology Drug Review (pCODR), the arms of the Canadian Agency for Drugs and Technology in Health that make recommendations about whether provincial governments should fund medicines in general (CDR) and oncology products in particular (pCODR). Specifically, this research examines whether there is an association between the positions that the groups take about the products under consideration and the receipt of money from companies.

## Methods

### Sources of data

Canada has no national drug formulary and as a result CDR makes recommendations to federal, provincial and territorial drug plans (except for Quebec) about whether to fund a unique drug-indication combination and pCODR does the same for oncology products. Briefly, CDR and pCODR accept applications from manufacturers and drug plans and then utilize expert panels [4, 5] that consider the clinical evidence, plus input from manufacturers, clinicians and patient groups in making their recommendations about whether the plans should list drugs for specific indications. CDR has been publishing the full submissions from patient groups since April 2013 and pCODR has been publishing summaries of submissions since January 2012. When patient groups make submissions they are asked to declare any conflicts of interest that they may have and these conflicts need to be stated for their views to be considered by CDR and pCODR. Prior to September 2017, groups making submissions to CDR could ask that their submissions be treated as confidential but in these cases their conflicts were still listed in the CDR Clinical Reports on the drug-indication. (The Institut national d’excellence en santé et en services sociaux (INESSS), the equivalent Quebec body, does not publish submissions from patient groups.)

Reports from CDR are available at https://www.cadth.ca/about-cadth/what-we-do/products-services/cdr/reports and from pCODR at https://www.cadth.ca/pcodr/find-a-review. Reports were included if they were labelled complete (CDR) or a final recommendation had been issued (pCODR) as of July 22, 2018 and if they included a submission from one or more patient groups. If two or more patient groups collaborated on a submission these were treated as separate submissions from each group if the groups had individual conflict declarations. Applications from manufacturers where they were requesting a reconsideration of a previous decision were included. CDR has a category called “Request for Advice” and reports in this category were not included as the patient group submission does not contain a conflict of interest statement.

### Information extracted from CDR and pCODR reports

From each report the following information was extracted: generic and brand name of drug, indication, company manufacturing the drug, the names of patient groups making submissions, conflict of interest statements from the patient groups, recommendations about funding and whether the recommendation was issued by CDR or pCODR. CDR issues three types of recommendations–list, list with criteria or conditions and do not list. Similarly, pCODR has three types of recommendations–fund, fund conditional on the drug being cost-effective or only for particular groups of patients and do not fund.

Only conflicts with either pharmaceutical companies or lobby groups representing pharmaceutical companies were recorded. Conflicts could be with the company marketing the drug under consideration or with other companies. Prior to August 31, 2017, CDR did not require groups to say when conflicts had occurred. Also prior to that date, patient groups could only make submissions to pCODR if they received funding from more than one company and if no single company provided more than 50% of the group’s operating funds [6]. From September 1 2017 onward, CDR and pCODR aligned their requirements for declaring conflicts, and groups were asked to list only conflicts with companies or organizations within the previous 2 years that may have a direct or indirect interest in the drug under review. In addition, after that time groups had to declare the amount of money that they had received from individual companies within four brackets: $0-$5,000, $5,001-$10,000, $10,001-$50,000 and >$50,000 [7].

### Analyses of information from CDR and pCODR reports

The number of individual drug-indication reports from CDR and pCODR were totaled along with the number of individual patient group submissions per drug-indication, whether the group did and did not have a conflict with the company marketing the drug or have any conflict at all, the total number of conflicts with drug companies for each individual submission and the total number of different submissions from each individual patient group.

The reports from CDR include the full patient group submission, although prior to August 31, 2017 CDR gave patient groups the option of refusing to publicly disclose their submissions, but in this situation it summarized patient group views in its Clinical Reports. If the submissions were public, extracts were taken from the submission. If more than one patient group made a submission about a drug-indication each submission was treated separately. If the full submissions were confidential, the CDR summaries were used. However, this was only possible if there was only a single submission since the CDR summaries did not attribute views to a particular group. Similar to the situation where CDR summarized confidential group submissions, if there was only a submission from a single patient group to pCODR then extracts from the summaries were used.

Two people, the author and CO a retired family physician, blinded as to any declarations of conflicts by the patient groups, independently read these extracts to determine whether the group’s view about the drug-indication was positive, neutral or negative. A priori, positive views were defined as ones that used words such as “effective” or “improvement”, negative views were defined as ones that used words such as “contraindicated” or “difficult to use.” Views that used neither of these terms nor their equivalent were classified as neutral. Disagreements were resolved by discussion. Patient groups were put into three categories–those with a conflict with the company marketing the drug, those without a conflict with the particular company but with conflicts with other companies and those with no conflicts at all. The distribution of views (positive, neutral, negative) of these three categories of patient groups were compared.

Patient group views about drug-indications were also compared with the recommendations from CDR and pCODR. For purposes of this comparison recommendations from CDR were collapsed into list (list + list with criteria) and don’t list and recommendations from pCODR were similarly dichotomized as fund (fund + fund conditional on cost-effectiveness) and don’t fund. Views from patient groups were dichotomized as positive or negative with a neutral view first being treated as positive and then as negative. These comparisons were done for all drug-indication recommendations except those for subsequent entry biologics (SEB) and separately for subsequent entry biologics. A SEB is a product that is similar to, and would enter the market subsequent to, an approved innovator biologic [8]. This group of drugs was chosen because patient groups may have been funded by companies marketing the originator biologic or the SEB and the source of the funding may have affected the view of the group.

Besides a final report, pCODR also issues preliminary reports with a funding recommendation. Patient groups are offered the opportunity to comment on those recommendations and give reasons for their comments. Comments are one of three types–agree, agree in part and disagree. If a patient group made a comment and reported the presence or absence of a conflict the comment was recorded along with the reasons that they gave to justify their comments. The distribution of the type of comment was compared for each of the three possible funding recommendations.

### Statistics

Kappa values were used to compare the scoring of views of patient groups by the two independent reviewers. Kappa scores measure whether there is more or less agreement between different evaluations than would be expected by chance. Levels of agreement were graded in accordance with the recommendations of Landis and Koch where < 0 indicates no agreement, 0–0.20 slight agreement, 0.21–0.40 fair agreement, 0.41–0.60 moderate agreement, 0.61–0.80 substantial agreement, and 0.81–1.0 almost perfect agreement [9]. The distribution of views of patient groups (positive, neutral, negative) about drug-indications under consideration was compared using a Chi square statistic. Agreement between views of patient groups and the recommendations from CDR and/or pCODR was compared using Fisher’s exact test. Prism 7.0d for Macintosh (GraphPad Software Inc.) was used for statistical testing.

### Patients and ethics

No patients were involved in this study and all data was publicly available and therefore ethics approval was not necessary.

## Results

There was a total of 222 reports for drug-indications– 114 from CDR and 108 from pCODR. There were 372 submissions from 93 different patient groups, 230 submissions for products considered by CDR (range 1–6 submissions per drug-indication) and 142 for pCODR products (range 1–3 submissions per drug-indication). Individual patient groups made between 1 and 15 separate submissions (S1 Table).

Groups declared a total of 1896 conflicts with drug companies and organizations representing drug companies in 324 (87.1% of the total number of submissions) individual submissions to both CDR (208 submissions and 1493 conflicts) and pCODR (119 submissions and 403 conflicts). The median number of conflicts per submission for CDR was 7 (interquartile range (IQR) 4,10) and for pCODR it was 1 (IQR 1,5). Overall there were 279 submissions (86.1% of all cases where the submission declared a conflict and 75.0% of the total number of submissions) where there was a conflict with the company marketing the product for a total of 1557 conflicts–CDR 164 submissions and 1164 conflicts, pCODR 115 submissions and 393 conflicts. In 48 submissions there was no conflict with the company marketing the product although conflicts with other companies were declared–CDR 44 submissions and 329 conflicts, pCODR 4 submissions and 16 conflicts. In 30 submissions no conflict with any company was declared (CDR 15 submissions and pCODR 15 submissions). Whether conflicts existed could not be determined for the remaining 15 submissions–CDR 7 submissions, pCODR 8 submissions (Table 1).

**Table 1:** Number of conflicts per patient group submission.

Status of conflict declaration in patient group submission		Number of patient group submissions (percent all submissions)		Total number of conflicts declared	
		Common Drug Review	panCanadian Oncology Drug Review	Common Drug Review	panCanadian Oncology Drug Review
No. of submissions with conflicts declared		208 (87.8)	119 (83.8)	1493	403
	Conflict with company marketing drug	164 (69.2)	115 (81.0)	1164	393
	No conflict with company marketing drug	44 (18.6)	4 (2.8)	329	16
No. of submissions declaring no conflict		15 (6.3)	15 (10.6)	0	0
No. of submissions where conflicts not known		7 (3.0)	8 (5.6)		
	Name of conflicted company not given	6 (2.5)	8 (5.6)		
	Conflict statement missing	1 (0.4)	0 (0)		
**Totals**		**237**	**142**		

The degree of granularity in the declaration of conflicts varied considerably, especially for patient groups who made submissions to CDR. Some did not state the time period when the conflicts occurred, whereas others gave specific time periods but those time periods varied from 1 year to 12 years. In other cases, groups declared that they had a conflict with one company and a number of unnamed other companies. There were 20 submissions (8 different patient groups) where the conflict declaration stated how much of the group’s total budget came from donations from pharmaceutical companies, but many of the 8 groups were inconsistent in providing that information (S2 Table). In the case of Myeloma Canada, the Asthma Society of Canada and Tuberous Sclerosis Canada donations from companies were a substantial portion of the organization’s budget– 36%, 20% and <20%, respectively, but for Cystic Fibrosis Canada, Multiple Sclerosis Society of Canada and Foundation for Fighting Blindness, the amounts were very small– 1.5% - 2.0%, <2%, 0.9%, respectively. In two other submissions, patient groups declared their conflicts but did not seem to see any value in making those declarations and included the following statement: “We do not see the purpose of asking how much money has been contributed by any entity that may have an interest in this.”

(https://www.cadth.ca/sites/default/files/cdr/relatedinfo/SR0522_Galafold_Patient_Input.pdf) Occasionally groups gave vague statements about how the money that they received from companies was used, e.g., for research or educational events, but 265 of the 324 (81.7%) declarations only named the companies that had donated money to the groups. In addition, after September 1, 2017 21 submissions listed grants from companies in one of four brackets: $0-$5,000–18 companies, $5,000 - $10,000–12 companies, $10,001 - $50,000–20 companies, >$50,000–43 companies. (Some submissions named more than one company, some companies gave money to more than one group.)

There were 268 submissions to CDR and pCODR, excluding submissions involving subsequent entry biologics, where patient groups declared a conflict with the company making the product (216 submissions), the absence of a conflict with the company (29 submissions) or no conflict at all (23 submissions) and where the groups expressed a view about the drug-indication being considered. Inter-rater reliability (Kappa) in scoring the views of patient groups was 0.4662 (95% CI 0.3040, 0.6284). The relatively low agreement between the two reviewers was due to initial confusion about what constituted a “neutral” opinion (34 of 36 disagreements). Table 2 gives examples of positive, neutral and negative views from patient groups.

**Table 2:** Examples of patient group positive, neutral and negative views about a drug-indication.

Group view	Examples of statements
**Positive**	• Patients are seeking effective treatment options and patients who had taken vismodegib reported that their condition had stabilized without progression, many for the first time in their lives. • Patients reported that the drug brought their disease under control, often quite quickly, and made them feel much as they had before their diagnosis of CLL.
**Neutral**	• Input from CCSN indicated that patients would like nivolumab to reduce their side effects from their current treatments, stop disease progression, control their symptoms, and be accessible. • Patient respondents reported that compared with other therapies, trifluridine-tipiracil had fewer side effects overall and better QoL; however, they also noted issues with blood counts and fatigue.
**Negative**	• Despite the potential of this drug to treat a variety of patients, drug-drug interactions also limits its usefulness. In particular, several common HIV medications are contraindicated for use with glecaprevir/pibrentasvir. • Xolair injections are hard to access–they must be done in a trained clinic during office hours. For me this is over an hour from my home and I work full time. Injections must be booked monthly with fairly limited flexibility. All other medications are more easily accessed.

Groups had positive views in 242 (90.3%) submissions, neutral in 24 (9.0%) and negative in 2 (0.7%) (Table 3). Table 3 also shows that views are consistently positive and that the distribution of views–positive, neutral, negative–is the same regardless of whether groups have a conflict with the company marketing the drug, a conflict with other companies or no conflict at all, p = 0.3117, Chi square.

**Table 3:** Distribution of patient group views about drug-indications under consideration*.

Conflict	View of patient group		
	Positive	Neutral	Negative
**With company marketing drug**	191	23	2
**With other companies**	29	0	0
**No conflict**	22	1	0
**Total**	242	24	2

*Excluding submissions about subsequent entry biologics.

Distribution of views by conflict status not statistically different, p = 0.3117, Chi square

The comparison between views of patient groups and CDR and/or pCODR recommendations was analyzed grouping neutral views from groups with negative ones. (Grouping neutral views with positive ones did not change the outcome of any of the comparisons, results not shown.) Fisher’s exact test shows no difference between the views of patient groups about drug-indications and the combination of CDR and pCODR recommendations (p = 0.78). Similarly, there was no difference when views of patient groups were compared separately with CDR or pCODR recommendations (p = 0.38 and p = 0.17, respectively, Fisher’s exact test) (Table 4). Finally, regardless of whether patient groups had a conflict with a company making the drug, a conflict with another company or no conflict, there was no statistical difference between their views and the combination of CDR and pCODR recommendations (p = 0.99, p = 0.20, p = 0.99, respectively, Fisher’s exact test) (Table 4).

**Table 4:** Comparison of views of patient groups with recommendations from Common Drug Review and panCanadian Oncology Drug Review*†.

Comparison	Fisher’s exact test (p value)
Patient group views and recommendations from CDR + pCODR	0.78
Patient group views and recommendations from CDR	0.38
Patient group views and recommendations from pCODR	0.17
Patient groups with conflict with company making drug and recommendations from CDR + pCODR	0.99
Patient groups with conflict with other companies and recommendations from CDR + pCODR	0.20
Patient groups with no conflict and recommendations from CDR + pCODR	0.99

*Excluding submissions about subsequent entry biologics

†Neutral views grouped with negative views

There were 22 submissions about subsequent entry biologics, but in one case it was not possible to determine if the group had a conflict and in a second case the view of the group was not available. In the other 20 cases the distribution of views did not show any clear pattern regardless of whether the groups had a conflict with the submitting company, the originator company, both types of companies or no conflict with any companies (Table 5). The small numbers precluded any meaningful statistical analysis.

**Table 5:** Subsequent entry biologics (SEB)–distribution of views of patient groups.

Conflict with:	Positive	Neutral	Negative
**Originator and SEB company**	1	7	2
**Neither**	1	0	0
**SEB company only**	1	0	0
**Originator company only**	2	3	3

Seventy (62.5%) submissions from patient groups (out of a total of 112 submissions where the group declared a conflict with the company marketing the drug) commented on pCODR preliminary recommendations. In 19 cases, pCODR did not recommend funding and in 17 of those 19 (89.4%), patient groups disagreed with the decision. Otherwise, patient groups either agreed or agreed in part with a decision to either fund or fund conditional on the drug-indication being cost-effective in 48 of 51 cases (Table 6). In the one case where a patient group disagreed with the recommendation to fund, it was because the group felt that the recommendation meant that certain groups of patients would not be eligible to receive the drug and this took away patients’ choice. The distribution of patient group responses in relation to the three funding recommendations from pCODR was statistically significantly different, p < 0.0001 (Fisher exact test). There were 10 submissions containing comments from groups with no conflicts with the company marketing the drug, too few to allow for any meaningful statistical analysis.

**Table 6:** Patient group response to preliminary recommendation from panCanadian Oncology Drug Review.

		Preliminary recommendation from panCanadian Oncology Drug Review		
		Fund	Fund with conditions or criteria	Do not fund
Response from patient group*	**Agrees**	4	33	0
	**Agrees in part**	1	10	2
	**Disagrees**	1	2	17

*Where patient group declared a conflict with company marketing product

p < 0.0001, Fisher exact test

## Discussion

When patient groups made submissions to CDR and pCODR about drug-indications, 75% of the time these groups declared a conflict with the company marketing the drug. The total of 1896 conflicts is a minimum, as some groups only listed companies that had a direct or indirect interest in the drug under consideration, whereas other groups made more comprehensive declarations. Other times, groups declared conflicts with one company and said they had additional conflicts but did not name the companies. Finally, there was no independent search for additional conflicts that groups may have had.

Patient groups’ views about products were positive just under 90% of the time and whether or not groups had a conflict with the particular company making the product under consideration, a conflict with another company or had no conflict at all did not make a difference in the distribution of their positive, neutral or negative views. At the same time, the views of patient groups were not statistically different from the recommendations from CDR and/or pCODR regardless of the presence or absence of conflicts.

One explanation for the almost uniform positive recommendations from all patient groups is that they may be motivated to make a submission because they feel strongly that the drug-indication will have a positive benefit for the patients that they represent and having a conflict plays no role in coming to this conclusion. This view is supported by the lack of any statistical difference between views of patient groups and the recommendations from CDR and/or pCODR. pCODR’s refusal prior to September 1, 2017 to accept submissions from groups likely to be the most affected by conflicts–those receiving funding from only a single company and those where a single company contributed more than 50% of their budget–may have skewed the results. However, there is no publicly available data about whether any patient group was actually denied participation because of this provision. There were too few submissions regarding subsequent entry biologics to draw any conclusions about whether the presence or absence of conflicts was associated with the views of patient groups.

The presence of a conflict with a company making the drug under consideration may play a role in the views of patient groups in certain circumstances. When the preliminary recommendation from pCODR was not to fund a drug-indication, patient groups almost always (17 out of 19 cases) disagreed and all of the groups that disagreed had a conflict with the company making the drug. When the recommendation was to fund or fund conditional on the drug being cost-effective, groups were in agreement with the recommendation in 48 out of 51 cases. Patient groups may have disagreed with the preliminary recommendation not to fund a drug-indication because they felt that the recommendation did not fully reflect their views about treatment duration, side effects, adherence, independence, psychosocial quality of life and avoiding further disease [10].

This study does not prove that funding from pharmaceutical companies directs the views that patient groups have about whether drugs should receive public funding. However, industry funding does put patient groups in a conflict of interest situation, where their primary interest is in the welfare of the patients that they represent and a secondary interest is in the financial health of the companies that provide them with funding and that are marketing the drugs under consideration. The possibility that the position that patient groups adopt may be influenced by their funding is raised by the guide that Medicines Australia published for its membership. The guide says that companies that sponsor non-profit groups might find that such sponsorship increases their chances of getting their drugs publicly funded under Australia’s Pharmaceutical Benefits Scheme [11].

Batt has provided examples of how Canadian breast cancer patient groups altered their behaviour once they started to accept donations from drug companies [1]. After the National Institute for Excellence in Health and Social Services of Quebec recommended that the Ministry of Health not fund 4 oncology drugs due to cost-effectiveness concerns, the Coalition Priorité Cancer (CPC), a Quebec-based patient advocacy group, denounced the decision and lobbied extensively to reverse it and was successful for 3 of the 4 drugs. A subsequent investigation of the group concluded that its commitment to its patient-members did not appear to be optimal based on a number of observations, including the absence of a clear position or warning against the use of bevacizumab for breast cancer and the CPC’s focus on the issue of reimbursement of expensive, low-efficiency drugs [12].

Reports from other countries have documented an association between patient groups receiving industry funding and the positions that they take in areas of concern to drug companies. In the United States (US), the Centers for Medicare and Medicaid Services proposed a project aimed at lowering spending on the most costly treatments offered under Medicare Part B, the federal insurance plan that covers outpatient drug costs. The proposed reform was to study the effect of modifying reimbursement methods so as to decrease physicians’ incentives to administer the most expensive medications. In addition to drug companies and doctors’ groups, 147 patients’ groups signed letters opposing the project, 110 of which received funding from the pharmaceutical industry [13]. The Epilepsy Foundation which receives funding from 4 companies marketing medications for epilepsy and that has representatives from these 4 companies on its board, campaigned for bills introduced in US state legislatures that would make it harder for pharmacists to substitute generic drugs for brand name epilepsy medications [14]. A survey of patient and consumer groups in Europe found an association between receiving industry sponsorship and support for an expanded role for the pharmaceutical industry as an information provider to the public about the products that companies make [15].

There is no research into the number or percent of Canadian patient groups that receive funding from pharmaceutical companies, but two recent papers from the US have explored this issue. McCoy and colleagues [16] looked at 104 groups with annual revenue of at least $7.5 million (USD). Eighty-six (83%) reported receiving donations from the pharmaceutical industry and only 1 explicitly stated that it did not receive donations. A second survey of a random sample of 7,685 patient groups operating in the US found that just over two-thirds of the responding organizations reported receiving industry funding, with almost 12% getting more than half of their funding from industry [17]. The recently passed legislation in Ontario requiring the disclosure of all transfers of value to healthcare professionals, healthcare institutions and organizations and patient groups [18] should provide Canadian data about patient group funding, at least for groups based in Ontario, provided the newly elected Progressive Conservative government implements the legislation.

Many patient groups in North America accept industry funding, but the situation may be different in a couple of European countries. Batt’s interpretation (Sharon Batt, personal communication, August 18, 2018) of a report on health consumer and patient organizations in seven European Union countries [19] is that the relatively small number of health consumer and patient organizations in the Netherlands and Sweden (200–250 and 50–100, respectively) makes government funding there more feasible. The Ministry of Health in the Netherlands spends more than 40 million euros annually on these groups and only a limited number of groups in that country receive funding from pharmaceutical companies. In Sweden, almost all groups receive structural funding from the Ministry of Health and Social Affairs. Whether groups in these countries take different positions regarding industry interests compared to groups in countries that are more reliant on industry money has not been explored.

### Limitations

Conflicts could not be determined in 15 submissions. Patient groups that declared a conflict with the company marketing the drug only commented on preliminary pCODR recommendations in 62.5% (70 out of 112) submissions. How the other groups felt about the recommendations in other submissions is not known. Whether groups agreed with the final recommendation from pCODR is not known although it seems unlikely that they would have changed their views between the preliminary and final recommendations. There is no data about how patient groups felt about CDR recommendations. Because of the lack of uniformity in how patient groups declared their conflicts some groups may have had undeclared conflicts. There was an element of subjectivity in classifying patient groups’ views about a drug-indication which is why there was an a priori definition for positive, neutral and negative view and why duplicate independent coding of these views was undertaken. The evaluation of comments about drug-indications being considered by pCODR and in some cases by CDR were based on pCODR and CDR summaries rather than directly from the submissions by the patient groups. The main strength of this study is that it looked at the entire population of recommendations from CDR and pCODR where patient groups had expressed a view about the drug-indication being considered.

## Conclusion

The large majority of patient groups that make submissions to CDR and pCODR have conflicts with the companies marketing the drugs and have positive views about the products that they are commenting on. Whether this is an association or a cause and effect or whether conflicts even play a role in the views of patient groups have important policy implications for patient groups, governments and health technology assessment bodies. If there is a cause and effect, then patient groups need to consider whether they are serving their membership by accepting industry funding, governments need to think about working with patient groups to develop new sources of unbiased support and health technology assessment agencies need to consider the weight that they give to patient group input. Further research to try and resolve this question is urgently needed. In the meantime, both CDR and pCODR should adopt a precautionary principle approach and require groups to disclose all of their conflicts, state the percent of their total funding that comes from pharmaceutical companies and organizations representing companies, extend the reporting period for conflicts to 5 years prior to the date of submission and revert back to the pre-September 2017 position of pCODR and exclude groups that are funded by only a single company or that derive more than 50% of their revenue from a single company.

## Supporting information

S1 Table Number of submissions per patient group. (DOCX)

S2 Table Patient groups declaring what percent of budget came from donations from pharmaceutical companies. (DOCX)
