# Supplementary material for: Association between commercial funding of Canadian patient groups and their views about funding of medicines: An observational study
Source: PLoS One. 2019 Feb 15;14(2):e0212399. doi: 10.1371/journal.pone.0212399 (PMC6377138; doi:10.1371/journal.pone.0212399)
Supplement: S1 Table — (DOCX) [file pone.0212399.s001.docx]

**S1 Table. Number of submissions per patient group**

| **Number of patient groups** | **Number of submissions per group** | **Total number of submissions** |
| --- | --- | --- |
| 36 | 1 | 36 |
| 12 | 2 | 24 |
| 11 | 3 | 33 |
| 5 | 4 | 20 |
| 5 | 5 | 25 |
| 1 | 6 | 6 |
| 2 | 7 | 14 |
| 5 | 8 | 40 |
| 5 | 9 | 45 |
| 4 | 10 | 40 |
| 1 | 11 | 11 |
| 2 | 12 | 24 |
| 3 | 13 | 39 |
| 1 | 15 | 15 |
| **93** |  | **372** |
