# Supplementary material for: Association between commercial funding of Canadian patient groups and their views about funding of medicines: An observational study
Source: PLoS One. 2019 Feb 15;14(2):e0212399. doi: 10.1371/journal.pone.0212399 (PMC6377138; doi:10.1371/journal.pone.0212399)
Supplement: S2 Table — (DOCX) [file pone.0212399.s002.docx]

**S2 Table. Patient groups declaring what percent of budget came from donations from pharmaceutical companies**

| **Name of patient group** | **Number of submissions where declaration made** | **Percent of budget from pharmaceutical companies** | **Total number of submissions made by group** |
| --- | --- | --- | --- |
| Asthma Society of Canada | 2 | 20% | 4 |
| Crohn’s and Colitis Canada | 5 | 9.5%, 11% (different years) | 5 |
| Cystic Fibrosis Canada | 3 | 1.5%, <2% (different years) | 3 |
| Foundation for Fighting Blindness | 1 | 0.9% (calculated by author from figures given) | 1 |
| Lung Cancer Canada | 1 | 15%, 20% (different years) | 13 |
| Multiple Sclerosis Society of Canada | 5 | <2% | 7 |
| Myeloma Canada | 2 | 36% | 10 |
| Tuberous Sclerosis Canada | 1 | <20% | 1 |
